# *Pisum sativum* Defensin 1 Eradicates Mouse Metastatic Lung Nodules from B16F10 Melanoma Cells

**DOI:** 10.3390/ijms21082662

**Published:** 2020-04-11

**Authors:** Virginia Sara Grancieri do Amaral, Stephanie Alexia Cristina Silva Santos, Paula Cavalcante de Andrade, Jenifer Nowatzki, Nilton Silva Júnior, Luciano Neves de Medeiros, Lycia Brito Gitirana, Pedro Geraldo Pascutti, Vitor H. Almeida, Robson Q. Monteiro, Eleonora Kurtenbach

**Affiliations:** 1 Instituto de Biofísica Carlos Chagas Filho, Universidade Federal do Rio de Janeiro, Rio de Janeiro, RJ 21941-902, Brasil; vi_farma@biof.ufrj.br (V.S.G.d.A.); stephaniealexia@biof.ufrj.br (S.A.C.S.S.); capaulinha1@gmail.com (P.C.d.A.); jeninowatzki@gmail.com (J.N.); nlju1@biof.ufrj.br (N.S.J.); lnmedeiros@gmail.com (L.N.d.M.); pascutti@biof.ufrj.br (P.G.P.); 2 Instituto de Bioquímica Médica Leopoldo de Meis, Universidade Federal do Rio de Janeiro, Rio de Janeiro, RJ 21941-902, Brasil; vhluna@bioqmed.ufrj.br (V.H.A.); robsonqm@bioqmed.ufrj.br (R.Q.M.); 3 Instituto de Ciências Biomédicas, Universidade Federal do Rio de Janeiro, Rio de Janeiro, RJ 21941-902, Brasil; lyciabg@histo.ufrj.br

**Keywords:** metastasis model of B16F10 melanoma, *Pisum sativum* defensin 1 (*Ps*d1), anti-metastatic activity, glucosylceramide (GlcCer), cyclin F

## Abstract

*Ps*d1 is a pea plant defensin which can be actively expressed in *Pichia pastoris* and shows broad antifungal activity. This activity is dependent on fungal membrane glucosylceramide (GlcCer), which is also important for its internalization, nuclear localization, and endoreduplication. Certain cancer cells present a lipid metabolism imbalance resulting in the overexpression of GlcCer in their membrane. In this work, in vitroassays using B16F10 cells showed that labeled fluorescein isothiocyanate FITC-*Ps*d1 internalized into live cultured cells and targeted the nucleus, which underwent fragmentation, exhibiting approximately 60% of cells in the sub-G0/G1 stage. This phenomenon was dependent on GlcCer, and the participation of cyclin-F was suggested. In a murine lung metastatic melanoma model, intravenous injection of *Ps*d1 together with B16F10 cells drastically reduced the number of nodules at concentrations above 0.5 mg/kg. Additionally, the administration of 1 mg/kg *Ps*d1 decreased the number of lung inflammatory cells to near zero without weight loss, unlike animals that received melanoma cells only. It is worth noting that 1 mg/kg *Ps*d1 alone did not provoke inflammation in lung tissue or weight or vital signal losses over 21 days, inferring no whole animal cytotoxicity. These results suggest that *Ps*d1 could be a promising prototype for human lung anti-metastatic melanoma therapy.

## 1. Introduction

Plant defensins (PDs) belong to the superfamily of cationic rich antimicrobial peptides (AMPs) [1] and are produced by plants as part of their innate immunity [2]. To date, over 100 known plant defensin primary sequences have been described [3]. They display low primary sequence homology, apart from the cysteine residues that are common, two glycine residues (positions 12 and 33), and aromatic residues (positions 10 and 41) related to *Ps*d1 [4,5]. Nevertheless, the tertiary structures of PDs show a common cysteine stabilized αβ-fold (CSαβ-fold), characterized by one α-helix and three antiparallel β-sheets [1,6,7]. Because of their amphipathic characteristics, their ability to kill microorganisms can involve nonspecific electrostatic and hydrophobic interactions with positive plasmatic membranes [8,9]. Additionally, membrane-specific targets for some plant defensins have been described, such as phosphatidic acid, phosphoinositides, mannosylinositolphosphoryl-containing sphingolipids, and glycosphingolipids (GSLs) [5,8,10,11,12,13,14].

*Pisum sativum* defensin 1 (*Ps*d1) is a 46-amino acid residue plant defensin isolated from pea seeds that presents well-documented antimicrobial activity against several fungal species [4,7,15,16,17]. The mechanism of action proposed for *Ps*d1 antifungal activity includes its interaction with specific cell wall/membrane lipid targets, such as C8-desaturated and C9-methylated glucosylceramide (GlcCer), a fungal exclusive GSL, and ergosterol [8,11,16]. NMR spectroscopy analysis has demonstrated that *Ps*d1 activity is intimately linked to its structure, with glycine 12 anchored in the first loop (residues 7 to 17) and histidine 36 anchored in turn 3, which are important amino acid residues for interaction with the fungal plasmatic membrane [15]. Nevertheless, *Ps*d1 does not show good affinity to cholesterol-enriched lipid bilayers, such as those found in mammalian cell membranes, which suggests its high human therapeutic potential [5,16].

Previous results from our group using a yeast two-hybrid system revealed that cyclin F from *Neurospora crassa* could be an intracellular partner for *Ps*d1 [18]. At that time, we also described that *Ps*d1 was internalized in *Fusarium solani* planktonic cells, directing their cell cycle impairment and causing fungal endoreduplication. Furthermore, we showed that the entrance of fluorescein isothiocyanate FITC-labeled *Ps*d1 in *Candida albicans* cells was dependent on GlcCer synthesis [8], a dependence also shown for its full antifungal activity against *Aspergillus nidulans* [11].

In mammals, cyclin F is expressed during S phase and peaks during the G2 phase of the cell cycle, which is considered an emerging factor in genome maintenance [19,20]. Cyclin F is also known as F-box only protein 1 (FBXO1) with an F-box domain required for binding to Skp1. Skp1 recruits Cul1 (and RBX1 with Cul1), forming the SCF ubiquitin ligase machinery that recruits the E2 ligase for ubiquitylation of target substrates. It utilizes a hydrophobic patch within its cyclin box domain, also known as the WD repeat domain, to bind the CY motif (RxL), also known as cyclin binding domain, in the substrates following their ubiquitylation and degradation as a ribonuclease. Various cyclin F substrates have been identified in the last decade, such as ribonuclease RRM2 [21], in order to ensure genome stability and efficient DNA repair and synthesis [19]. Recently, Clijsters and colleagues showed that the three activators of the E2F family of transcription factors, E2F1, E2F2, and E2F3A, key regulators of the G1/S cell transitions, interact with the cyclin box of cyclin F, resulting in their degradation and impairment in cell fitness [22]. The carboxy-terminal region of cyclin F is the regulatory module that controls its nuclear and centrosome localization as well as its abundance during the cell cycle and following genotoxic stress.

More recently, new functions have been reported for AMPs, including chemotactic, immunomodulatory, oncolytic, and mitogenic activities, among others [9,23,24]. Indeed, some host defense peptides that selectively target cancer cell membrane components have excellent tumor tissue penetration and thus can reach the sites of both the primary tumor and distant metastases [25]. However, to date, only a few plant defensins have been reported to exhibit cytotoxic activity towards cancer cells in vitro [26].

It is well known that cancer cells suffer lipid metabolic reprogramming [27] that can lead to plasmatic membranes enriched with negatively charged phospholipid phosphatidylserine (PS), as previously reported [28] in melanoma cells when compared to non-neoplastic cells. Additionally, primary cultures and metastases in addition to other cancer types expose PS [29], in contrast to the normally neutral outer leaflet of the plasma membrane.

More specifically, cancer cells suffer dysregulation of sphingolipid metabolism, and increased expression of glucosylceramide synthase and the accumulation of glucosylceramide (GlcCer) in multidrug-resistant tumor cells have been described [30,31,32]. GlcCer is a neutral sphingolipid composed of a sphingoid base (or LCB, long chain base), a fatty acid chain and a glucose residue. It is found in most fungi, except in *Saccharomyces cerevisiae* and *Candida glabrata* [33] and is conserved in higher eukaryotes, such as plants and mammals. They are essential for cellular structural integrity and regulating the fluidity of the lipid bilayer and are involved in cell proliferation [34,35], differentiation [27,36], and oncogenic transformation [37,38].

The properties described so far have classified *Ps*d1 as a putative candidate for the development of a prototype for cancer therapy, representing a novel family of oncolytic agents that can discriminate between the neutral surfaces of non-cancerous cells and the negatively charged surfaces of cancer membranes, being cytotoxic towards a broad spectrum of malignant cells without impairing normal body physiological functions [26,39].

Thus, the main goal of this work was to test the cytotoxic effects of *Ps*d1 against cancer cells in vitro and in vivo using a mouse B16F10 lung metastatic model. We were able to demonstrate that *Ps*d1 decreased the viability of several cancer cells in vitro. Confocal images showed that *Ps*d1 caused permeabilization and was internalized in live cells, localizing to the nucleus. The participation of the protein cyclin F as an intracellular partner was reinforced by surface plasmon resonance (SPR) analysis and molecular docking when it was possible to detect several points of contact between these proteins. For the first time, the eradication of mouse metastatic B16F10 cell lung nodules by the plant defensin *Ps*d1 was successfully recorded.

## 2. Results

### 2.1. Psd1 Presents Selective Cytotoxic Effects against Tumor Cells In Vitro

**Figure 1:**
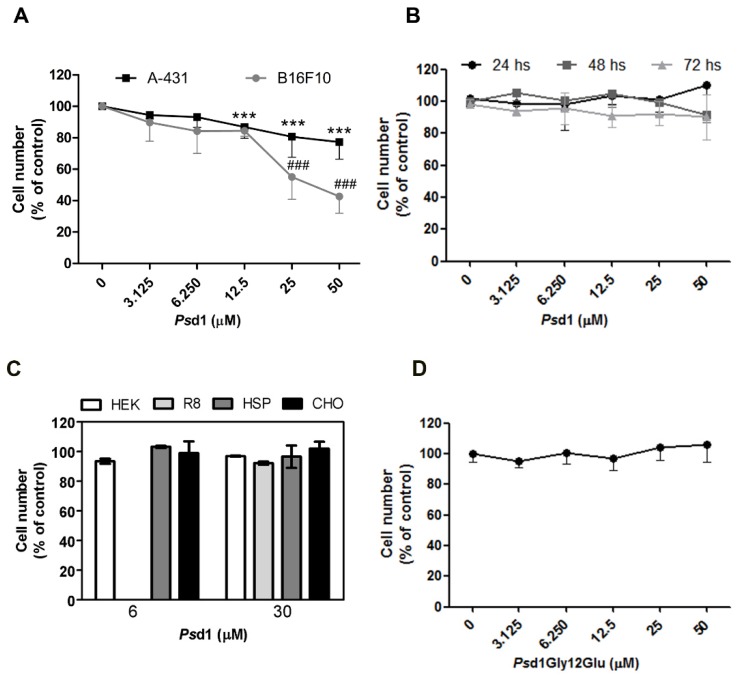
*Ps*d1 has cytotoxic activity against tumor cells but not against healthy cells in vitro by the MTT assay. Cells lines (5 × 10^4^ cells/well) were treated with different concentrations of *Ps*d1 (3.125, 6.25, 12.5, 25, and 50 µM). Data were normalized against the untreated control, which was arbitrarily assigned as 100% cell viability. (**A**) A-431 and B16F10 cells were treated with *Ps*d1 for 24 h. Both tumor cell lines had a significant reduction in viability in a dose-dependent manner. (**B**) Healthy Beas-2B cells were treated with *Ps*d1 for 24, 48, and 72 h. No significant difference was observed at any time tested. (**C**) Cell viability of different mammalian health lineages by (lactate dehydrogenase) LDH release after incubation with *Ps*d1 for 3 h. Values are the mean ± SEM of two experiments performed in triplicate. *Ps*d1 at concentration of 6 µM was not tested against R8 cells. (**D**) B16F10 cells were incubated with *Ps*d1 Gly12Glu for 24 h. The mutant did not show cytotoxic activity against this lineage. (**A**,**B**,**D**) Values are the mean ± SEM of two independent experiments performed in triplicate. Statistical significance was determined using one-way ANOVA with Dunnett’s multiple comparison test compared to cells without treatment. *** *p* < 0.001 for A431 cells in the presence of 12.5 µM, 25 µM, or 50 µM *Ps*d1 and ### *p* < 0.001 for B16F10 cells in the presence of 25 µM or 50 µM *Ps*d1.

To investigate the cytotoxic activity of *Ps*d1 on tumor cell viability, MTT-based colorimetric assays were performed using murine skin melanoma (B16F10), human epidermoid carcinoma (A-431), and healthy (Beas-2B, HEK, R8, HSP, and CHO) cell lines (Figure 1). Cell viability was detected by the ability of viable cells to transform yellow tetrazolium salt into purple formazan crystals [40]. As shown in Figure 1A, *Ps*d1 significantly inhibited the viability of both types of tumor cells in a dose-dependent manner after 24 h of treatment. A-431 cells had decreased growth in the presence of 12.5 µM peptide, reaching a maximum reduction of 20% with 50 µM peptide. B16F10 cells were reduced to approximately 50% and 60% when treated with 25 and 50 µM of the peptide, respectively. In contrast, when *Ps*d1 was incubated with healthy human bronchial epithelial cells Beas-2B (Figure 1B), HEK-293, R8, HSP-2, and CHO (Figure 1C) cells, no alteration in cell viability was observed even after 72 h of exposure in the case of Beas-2B cells (Figure 1B).

The conserved Gly12 residue in defensins [1] is crucial for *Ps*d1 antifungal activity [15]. *Ps*d1 was more effective against B16F10 cells, and therefore this lineage was chosen to evaluate the importance of this residue in antitumor activity. For this, *Ps*d1 Gly12Glu, with a glutamic acid at position 12 [15], was incubated with B16F10 tumor cells for 24 h using the same previous concentrations (Figure 1D). The mutant peptide was not able to interfere with B16F10 viability at any of the concentrations tested, which is different from the results observed with native *Ps*d1 (Figure 1A).

### 2.2. Insights into the Action Mechanism of Psd1 Antitumor Activity

#### 2.2.1. Psd1 Permeabilizes the Plasma Membrane and Induces Apoptosis in B16F10 Cells

**Figure 2:**
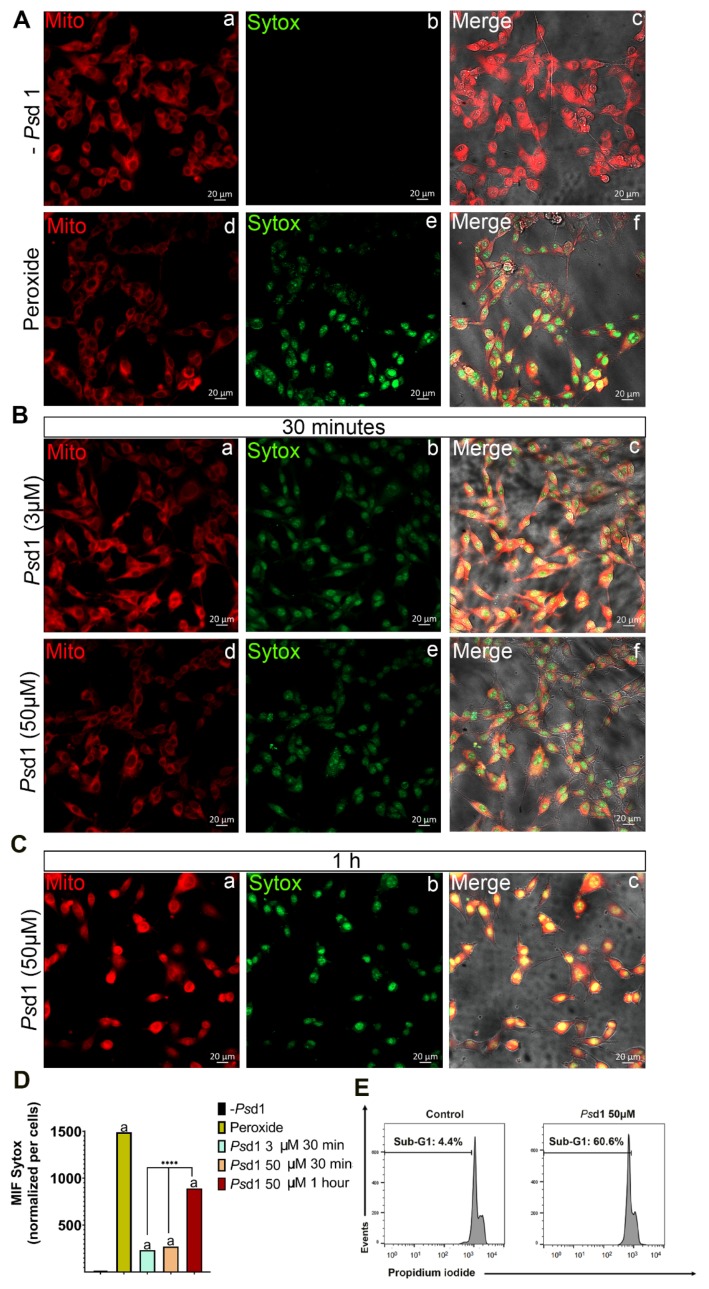
*Ps*d1 promotes B16F10 live cell membrane permeabilization as monitored by SYTOX Green fluorescence. (**A**) *Ps*d1 untreated and hydrogen peroxide-treated B16F10 cells. (**B**) A total of 1.5 × 10^4^ B16F10 cells were incubated with 3 or 50 µM *Ps*d1 for 30 min. (**C**) 1.5 × 10^4^ B16F10 cells were incubated with 50 µM *Ps*d1 for 1 h. All experiments were performed at 37 °C. Nucleus staining is shown in green (SYTOXGreen), and mitochondria are shown in red (MitoTracker Red CMXRos). Images are shown at 20× magnification. (**D**) Mean fluorescence intensities (MIF) of SITOXGreen fluorescence signals showed in (**A**, b,e), (**B**, b,e), and (**C**, b) per cell number are time-dependent. ****, *p* < 0.0001 treatment with 50 µM of *Ps*d1 for 1 hour when compared with treatments with 3 and 50 µM of *Ps*d1 for 30 min; letter a, *p* < 0.0001 treatments with peroxide and *Ps*d1 when compared with the absence of *Ps*d1 using one-way ANOVA and Bonferroni’s multiple comparison test. (**E**) *Ps*d1 induces apoptosis in B16F10 cells. B16F10 cells were treated with 50 µM *Ps*d1 for 24 h and labeled with propidium iodide, and the DNA content was analyzed by flow cytometry for the sub-G0/G1 profile. At least two independent experiments were performed.

Confocal fluorescence microscopy was performed to detect whether *Ps*d1 promotes B16F10 cell death by membrane permeability and apoptosis induction (Figure 2). For this, a nuclear dye impermeable to the plasma membrane, SYTOXGreen (SG), was used to monitor membrane integrity changes caused by *Ps*d1. Additionally, mitochondrial viability was monitored with MitoTrackerCMRos dye accumulation, which is dependent upon membrane potential. In the absence of *Ps*d1 (Figure 2A, a–c), the existence of attached well-formed cells with projections and an intact whole plasma membrane was observed. Further, intense MitoTrackerCMRos red marker indicated that the mitochondrial membrane potential was normal [41] and that mitochondria were metabolically active (Figure 2A, a). As expected, no SYTOXGreen fluorescence was detected (Figure 2A, b).

Contrarily, treatment with 3 µM and 50 µM *Ps*d1 for 30 min caused the entrance of SYTOXGreen into the cell, resulting in intense green nuclear staining (Figure 2B, b/e). The green fluorescence signal increased significantly when cells were treated with 50 µM for 1 h (Figure 2C, b), as observed in the presence of hydrogen peroxide (Figure 2A, e), indicating that the promotion of membrane damage by *Ps*d1 was time-dependent. When SYTOXGreen fluorescent signals were normalized by cell number it was possible to confirm a very significantly increase in treatment with 50 µM of *Ps*d1 for 1 h when compared with treatments with 3 and 50 µM of *Ps*d1 for 30 min. (****, *p* < 0.0001) (Figure 2D).

This effect was accompanied by a lower mitochondria red marker, mainly in the presence of 50 µM Psd1 for 30 min (Figure 2B, e). Surprisingly treatment with 50 µM *Ps*d1 for 1 h, that displayed higher SYTOX permeability, showed a brighter red fluorescence signal, probably due to the superposition of SYTOXGreen and MitoTrackerCMRos dye signals inside the cells (Figure 2C, a). In addition, in this situation B16F10 cells started to lose their adhesion capacity, assuming a less spread shape (Figure 2C, c). Together, these results suggest that *Ps*d1 altered the biophysical properties of plasmatic membrane of the tumor cells, which could be accompanied by intracellular death signaling events such as oxidative stress of mitochondria. However, more investigation about the mitochondrial effect caused by *Ps*d1 treatment must be performed.

Also B16F10 cells treated with 50 µM *Ps*d1 exhibited a high proportion of cells with sub-G0/G1 DNA content (~60%), as shown by flow cytometry (Figure 2D), which is indicative of cells undergoing DNA fragmentation. Internucleosomal DNA fragmentation is one of the hallmarks of apoptosis and is frequently used as a criterion for its detection [42]. Thus, these data corroborate the findings obtained in the MTT assay (Figure 1A), suggesting that the decrease in cell viability of *Ps*d1-treated B16F10 cells is associated with the induction of apoptosis.

#### 2.2.2. *Ps*d1 Internalizes towards the Nucleus

**Figure 3:**
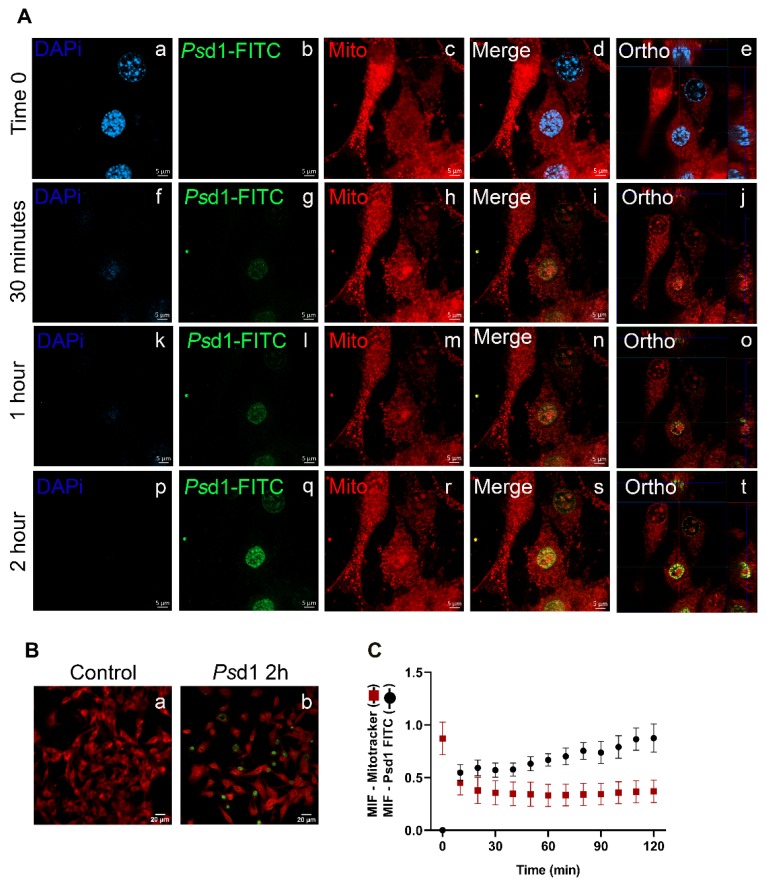
Fluorescein isothiocyanate (FITC)-*Ps*d1 was localized in the B16F10 live cell nucleus. (**A**) B16F10 cells (1.5 × 10^4^) were initially pre-stained with DAPI and MitoTracker Red CMXRos followed by the addition of 9 µM *Ps*d1-FITC. Images were acquired in real time every 10 min for two hours at 100× magnification. (**B**) B16F10 cells in the absence of *Ps*d1 (control time zero) and in the presence of 9 µM *Ps*d1 for two hours (*Ps*d1 2 h) at 10× magnification. (**C**) The mean fluorescence intensity (MIF) of MitoTracker and FITC-*Ps*d1 labeled cells measured over time. Average values with standard deviation (SD) of two independent experiments are reported.

The entrance of *Ps*d1-FITC in the B16F10 live cells in real time was observed using spinning disk confocal fluorescence microscopy (Figure 3). Before the addition of *Ps*d1 (time zero), B16F10 cells showed good plate adherence with nuclei well stained in blue (DAPI) and several mitochondria in red (MitoTracker Red CMXRos) (Figure 3A, a/c-d), indicating cell viability and normal mitochondrial transmembrane potential. No background concerning FITC signal was detected (Figure 3A, b). Then, 9 µM *Ps*d1-FITC was added, and the same cell field was photographed for 2 h. After 30 min, it was possible to detect few and faint green fluorescence signals in the nuclei, suggesting the entrance of *Ps*d1 in those cells (Figure 3A, g). The relative fluorescence of *Ps*d1-FITC increased after 1 h (Figure 3A, l). *Ps*d1-FITC fully accumulated in the nucleus after 2 h of treatment, where intense bright green fluorescent points were detected (Figure 3A, q). The nuclear location of *Ps*d1 was confirmed by merging the FITC and DAPI signals detected in merge and orthogonal views, as shown in the right panels (Figure 3A, i, n, s (merge) and j, o, t (ortho)). Moreover, over time, it was possible to detect a decrease in the red fluorescence intensity at real time (Figure 3A, c, h, m and r).

Pictures showing B16F10 cells marked with MitoTracker Red CMXRos and FITC-*Ps*d1 for 2 h in a smaller order of magnitude were shown in Figure 3B. In this case it was possible to have an overview of the *Ps*d1 internalization phenomena in a larger number of cells. Quantification of the regions of interest (ROI) relative to the mean fluorescence intensity (MFI) from the intensity of the pixels revealed that simultaneously, the red fluorescence decreased and the green fluorescence increased (Figure 3C). Together, these findings plus permeability results shown in Figure 2 strongly suggested that the *Ps*d1 entrance caused loss of membrane barrier function, compromising the cell cycle and mitochondrial roles. However, it is not clear the order in which these events happened, that is, does the peptide need to interact with nuclear targets and then trigger apoptosis events, collapsing the mitochondria, or this occur just after membrane interaction? Additional experiments will be done to answer these questions.

#### 2.2.3. *Ps*d1 Entrance in B16F10 Cells is Glucosylceramide-Dependent

**Figure 4:**
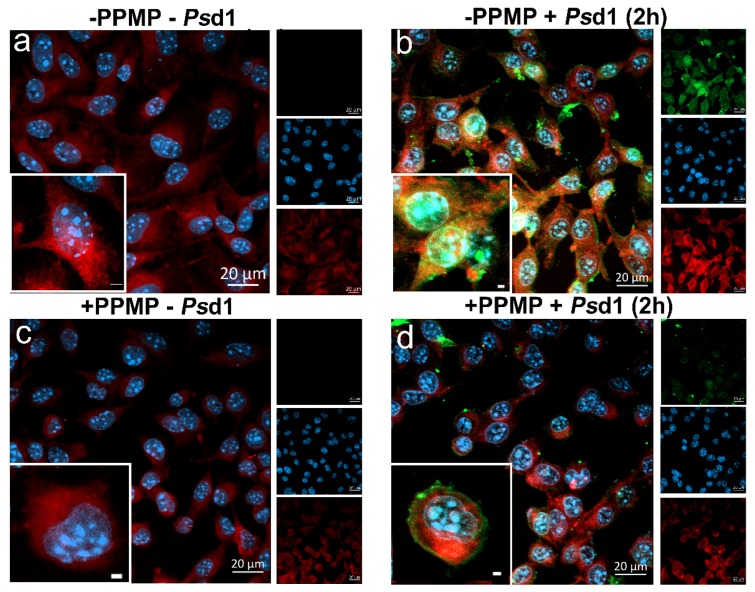
GlcCer reduction inhibits the entrance of FITC-*Ps*d1 into B16F10 cancer cells. B16F10 cells (1.5 × 10^4^) were incubated with or without 20 µM PPMP for 1 h followed by treatment with or without 9 µM FITC-*Ps*d1 for 2 h. Fixed cells were stained with Vybrant™ Alexa Fluor™ 594 and DAPI. (**a**) B16F10 cells without treatments (−PPMP, −*Ps*d1); (**b**) B16F10 cells treated with *Ps*d1 for 2 h (–PPMP, +*Ps*d1 (2 h)); (**c**) B16F10 cells treated with PPMP (+PPMP, −*Ps*d1); (**d**) B16F10 cells treated with PPMP plus *Ps*d1 (+PPMP, +*Ps*d1 (2 h)). Pictures were acquired at 40× magnification. Inset zoom images are at 100× magnification. At least two experiments were performed independently.

We evaluated whether glucosylceramide (GlcCer) could impact the entrance of *Ps*d1 in B16F10 cells by reducing its amount on the plasmatic membrane using DL-threo-1-phenyl-2-palmitoylamino-3-morpholino-1-propanol (PPMP) (Figure 4). PPMP is a well-studied glucosylceramide synthase inhibitor [30,32,43,44,45] with several reports about its action in B16 melanoma cells [30,46]. The amount of GlcCer in the plasmatic membrane was determined using the red cholera toxin subunit B (CT-B) compound [47,48,49], which binds specifically to raft domains, followed by treatment with an anti-CT-B antibody labeled with Alexa Fluor 594. As seen in Figure 4c, cells treated with PPMP showed a significant reduction in the bright red signal (Figure 4c, +PPMP, −*Ps*d1) relative to that observed in PPMP-free cells (Figure 4a, −PPMP, −*Ps*d1).

As GlcCer is the majority neutral GSL (87% of total) in B16F10 cells [50] and one of the major components of lipid rafts [51,52] we do believe that the diminished red fluorescent signal detected in the presence of PPMP could reflect the decrease of glucosylceramide in B16F10 cell membranes.

The image in Figure 4b (−PPMP, +*Ps*d1 (2 h)) clearly showed that in the absence of PPMP, FITC-Psd1 was internalized into B16F10 cells being localized in the cells nuclei, confirmed by the superposition between green and blue dyes. Meanwhile when B16F10 cells were treated with PMPP plus 9 µM *Ps*d1 for 2 h (Figure 4d, +PPMP, +*Ps*d1 (2 h)), a different profile was observed. Inthis new situation the nuclear FITC-*Ps*d1 green fluorescent signals were practically non-existent. *Ps*d1 was kept outside the cell and retained in the membrane, as noted by the green signals around the outside of the cell (Figure 4d, +PPMP, +*Ps*d1 (2 h), for details see the images insets with larger magnitude).

Together, the results using PPMP, a glucosylceramide synthase inhibitor, provided evidence that GlcCer actively participates in the *Ps*d1 interaction with the B16F10 membrane, internalization, and further death signaling mechanisms.

### 2.3. Psd1–Cyclin F Interaction by Surface Plasmon Resonance (SPR)

**Figure 5:**
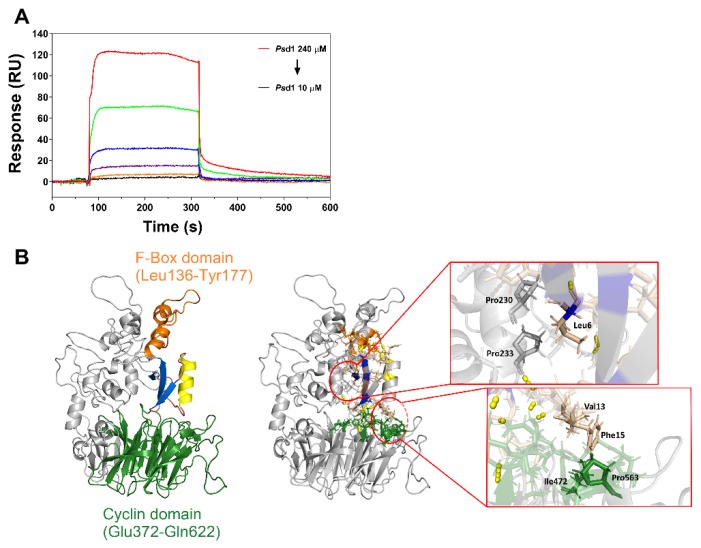
(**A**) The *Ps*d1-cyclin F interaction was confirmed by surface Plasmon resonance SPR. Sensorgram curves demonstrating the association and dissociation phases of immobilized cyclin F on the CM5 chip surface with different concentrations of *Ps*d1: 10 μM (black), 20 μM (orange), 40 μM (purple), 80 μM (blue), 160 μM (green) and 240 μM (red). Each experimental condition was performed at least twice. (**B**) The most favorable pose for the *Ps*d1-cyclin F interaction predicted by molecular modeling simulations. Left cartoon: Human cyclin F domains were designated in orange for the F-box domain and green for the cyclin domain (also known as the WD domain); *Ps*d1 secondary structures are shown in blue for β-strands and yellow for α-helices attached by pale pink loops(Protein Database Bank (PDB accession number 1JKZ). Right cartoon: Amino acid residues involved in the hydrophobic interface clusters are highlighted by red balloons and shown in the inset zoom images (Leu 6 from *Ps*d1 with Pro 230 and Pro 233 belonging to F-box domain of cyclin F, and Val 13 and Phe 15 from Psd1 with Ile 472 and Pro 563 belonging to the cyclin domain of cyclin F).

Considering that cyclin F is an intracellular target for *Ps*d1 in fungal cells [18], we evaluated the specificity of their interaction in real-time by SPR. Recombinant cyclin F was immobilized on the CM5 chip followed by a constant flow injection of *Ps*d1. The obtained sensorgram revealed the cyclin F/*Ps*d1 interaction as a function of *Ps*d1 concentration, indicating a dose-dependent effect (Figure 5A). Increases in plasmon resonance signals (RU) due to protein–protein binding were observed 80 s after *Ps*d1 injection. This response indicated a very fast association phase that reached a plateau until the injection ended. RU responses enhanced from zero to 2.5, 3.6, 11.5, 26, 64, and 119 RU when concentrations of 10, 20, 40, 80, 160, and 240 µM *Ps*d1 were tested, showing a closed, linear rise pattern response. A full dissociation phase was observed when the injection of *Ps*d1 ended, with all curves returning to baseline levels. The results of global fitting using the 1:1 Langmuir model allowed us to calculate an affinity constant (KD) of 1.5 mM, indicating that *Ps*d1 interacts with cyclin F in this cell-free system.

In parallel, in silico molecular docking was used to predict the best binding mode of *Ps*d1 to human cyclin F. This technique allows the generation of the most likely stable conformations and orientations, named poses, of *Ps*d1 within the cyclin F binding sites. The results are displayed in Figure 5B. *Ps*d1 fits into a small cavity of the cyclin F protein, with certain parts of its structure being in contact with the F-box and cyclin substrate recruitment domains (left image). Most of the *Ps*d1 defensin residues found in the interaction interfaces belonged to its loop regions (15 residues out of 24 found). Likewise, most of the cyclin F residues detected in the interface belonged to the F-Box and cyclin domains (21 residues out of 30 found). The peptide–protein complex was formed by the cumulative contribution of hydrogen bonds and electrostatic, hydrophobic and van der Waals interactions, although its stability over time could not be evaluated (Figure 5B, right image, Table S1 and Video S1).

At the interfaces, a total of 15 hydrogen bonds between *Ps*d1 and cyclin F were predicted. In the defensin structure, most of the participating residues belong to the loop regions (7 residues out of 11, namely, Leu6, Arg11, Gly12, Ala28, His29, Cys35, and Trp38), while in the cyclin F structure, a total of 11 residues took part in this type of interaction, with four residues belonging to the F-Box domain (Tyr147, Lys171, His175, and Tyr177) and another four residing in the WD-repeat domain (Lys470, Ile543, Glu545, and Arg546). Notably, both Arg11 of *Ps*d1 and Tyr147 of cyclin F make three distinct hydrogen bonds, which is more than any other residue in the complex. In addition to hydrogen bonds, the protein–peptide interfaces exhibited several hydrophobic contacts. In the case of *Ps*d1, all three engaging residues resided in loops (Leu6, Val13, and Phe15). With respect to cyclin F, four residues in total made meaningful contacts (Pro230, Pro233, Ile472, and Pro563). The inset zoom images in Figure 5B (right) represent the hydrophobic contacts between Leu6 from *Ps*d1 with prolines in positions 230 and 233 belonging to F-box domain of cyclin F, and Val 13 and Phe 15 from *Ps*d1, with Ile 472 and Pro 563 belonging to the cyclin domain of cyclin F. More details can be seen in Table S1 and Video S1.

### 2.4. Psd1 Impaired the Establishment of B16F10 Tumor Metastasis In Vivo

**Figure 6:**
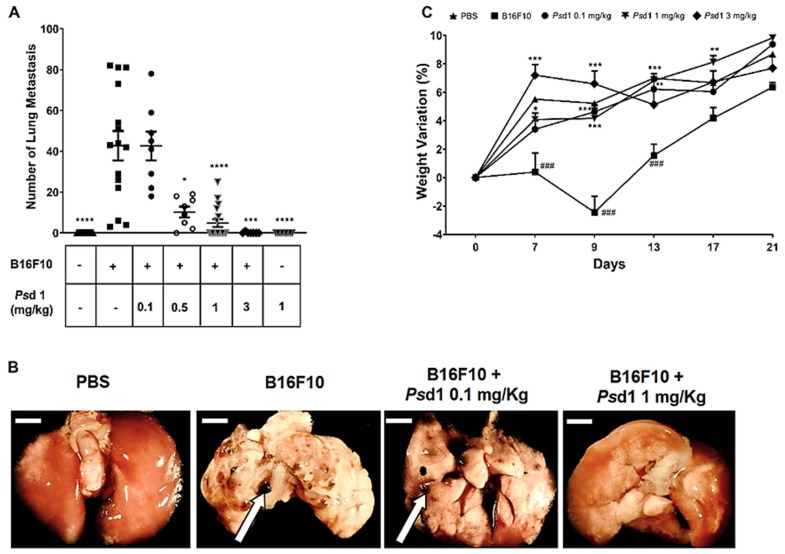
Protective effects of *Ps*d1 on a model of experimental lung metastasis in vivo. B16F10 mouse melanoma tumors were established by intravenous injection of 2.5 × 10^5^ B16F10 cells into C57BL/6 mice. (**A**) After 21 days, mice from each experimental group were sacrificed, and the number of visible lung metastatic nodules was quantified. Treatment of the animals inoculated with melanoma cells plus *Ps*d1 caused a dose-dependent reduction in the number of lung metastases. Black lines represent the mean ± SEM of two independent experiments analyzed by one-way ANOVA and Bonferroni’s multiple comparison test. **** *p* < 0.0001 B16F10 vs. phosphate buffer solution (PBS); **** *p* < 0.0001 B16F10 vs. *Ps*d1 1 mg/kg; **** *p* < 0.0001 B16F10 vs. just *Ps*d1 1 mg/kg (without B16F10 cells); *** *p* < 0.001 B16F10 vs. *Ps*d1 3 mg/kg; * *p* < 0.05 B16F10 vs. *Ps*d1 0.5 mg/kg. (**B**) Representative images of lung metastatic nodules from animals that received just PBS or B16F10 cells alone or together with 0.1 mg/kg or 1 mg/kg *Ps*d1. Bar = 2 mm. (**C**) Animal weights were acquired every two days. Weight variation was calculated as (weight on that day (weight of the first day × 100 − 100)). Only the days with higher weight differences between animals injected with only B16F10 cells and those that received cells plus *Ps*d1 or PBS alone are represented. All of the values represent the mean ± SEM of two independent experiments analyzed by two-way ANOVA. *** *p* < 0.001 B16F10 vs. PBS at 7, 9 and 13 weeks; *** *p* < 0.001 B16F10 vs. *Ps*d1 0.1 mg/kg at 9 weeks; ** *p* < 0.01 B16F10 vs. *Ps*d1 0.1 mg/kg at 13 weeks; *** *p* < 0.001 B16F10 vs. *Ps*d1 1 mg/kg at 9 and 13 weeks; ** *p* < 0.01 B16F10 vs. *Ps*d1 1 mg/kg at 17 weeks; * *p* < 0.05 B16F10 vs. *Ps*d1 1 mg/kg at 7 weeks; *** *p* < 0.001 B16F10 vs. *Ps*d1 3 mg/kg at 7 and 9 weeks.

A well-characterized mouse model of experimental lung metastasis by intravenous injection of B16F10 melanoma cells was used to evaluate the effect of *Ps*d1 in vivo. Different concentrations of *Ps*d1 were injected together with B16F10 cells into C57BL/6 mice via the tail vein. One group of animals received only 1 mg/kg *Ps*d1 to evaluate the possible toxicity of the peptide. Twenty-one days after injection, the mice were sacrificed and assayed. The lung nodule quantification from all groups is shown in Figure 6A, and representative lung images are shown in Figure 6B. Metastasis induction was successfully achieved, showing an average of 43 nodules in the lungs of mice that received only B16F10 melanoma cells (Figure 6A,B, *n* = 20). A significant reduction in lung metastasis colonization after treatment with *Ps*d1 was achieved in a dose-dependent manner beginning at the dose of 0.5 mg/kg. Treatment with 3.0 mg/kg *Ps*d1 completely abolished tumor development (Figure 6A, 0.14 ± 0.14 nodules). The number of pulmonary nodules decreased by 75% and 88% when 0.5 mg/kg (10.2 ± 2.6 nodules) and 1.0 mg/kg (4.82 ± 1.88 nodules) *Ps*d1 was used, respectively. Notably, animals that received either phosphate buffer solution (PBS) pH 7.4 or 1.0 mg/kg *Ps*d1 alone did not present lung nodules during the experimental period (Figure 6A).

The animal group that received just intravenous injection of PBS gained weight throughout the experimental period, as expected (Figure 6C). Meanwhile, animals from the B16F10 group, which were injected exclusively with B16F10 melanoma cells, showed a very large loss in body weight compared with the PBS group, being more pronounced on the ninth day. After this point, their weights partially recovered but remained lower than the other groups. Animals that received B16F10 cells plus 0.1, 1, and 3 mg/kg *Ps*d1 showed a similar pattern as those injected with PBS; that is, these animals gained weight throughout the assay. The results obtained in the presence of 1 and 3 mg/kg *Ps*d1 are consistent with less lung metastatic nodules occurring in these animals. However treatment with 0.1 mg/Kg was not effective in reducing the number of nodules but was effective in reducing their size when compared to animals that just received B16F10 cells (that not shown).We do believe that in this case these smaller nodules were not enough to trigger the pathways responsible to lose detectable weight.

It is important to note that mice treated with *Ps*d1 alone did not present changes in important behavior signals, such as changes in locomotion, piloerection, diarrhea, or mortality as preconized by ANVISA through the Guide for Conducting Non-Clinical Drug Safety and Toxicology Studies Required for Drug Development/Safety and Efficacy Assessment Management (GESEF 2013 version 2) when compared to animals that received PBS solution alone.

**Figure 7:**
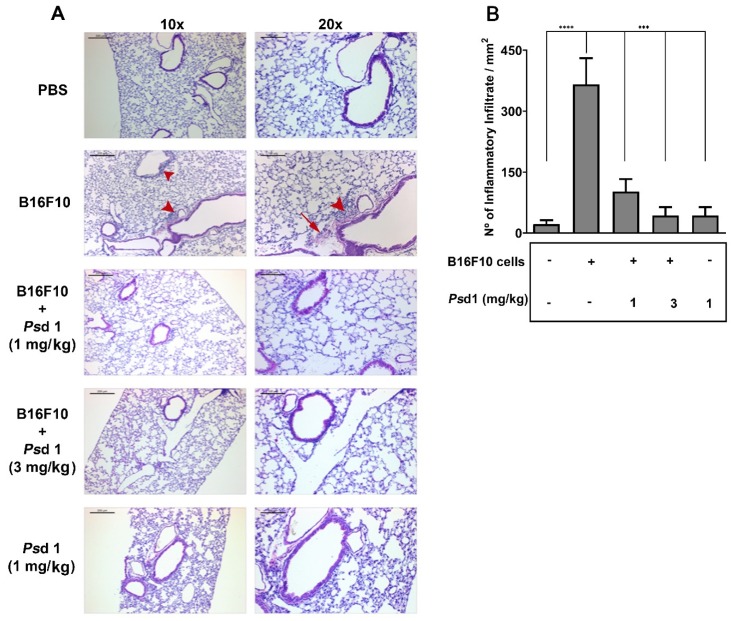
*Ps*d1 inhibited the appearance of inflammatory cells in lung tissues. Two animals from each group of the in vivo experiment were anaesthetized and submitted to transcardial perfusion for further histological procedures. The lungs were removed and prepared for hematoxylin and eosin (HE) staining as described in the Materials and Methods. (**A**) Representative images from the lungs of one animal from each experimental group. Animals injected with just B16F10 cells showed the presence of infiltrating cells around blood vessels (red head arrows) and fibrotic lesions (red arrow) compared to animals from the other groups. The left and right panels indicate 10× and 20× magnifications, and the bars represent 200 and 100 µM, respectively. (**B**) Quantification of inflammatory infiltration (± SEM) was obtained in six fields, corresponding to 90% of the tissue. A significant reduction in inflammatory cells was observed in animals treated with *Ps*d1. **** *p* < 0.0001 for B16F10 vs. PBS or *** *p* < 0.001 forB16F10 vs. *Ps*d1 1 mg/kg or B16F10 vs. *Ps*d1 3 mg/kg or *Ps*d1 1 mg/kg alone. Values represent the mean ± SD of two independent experiments analyzed by one-way ANOVA and Bonferroni’s multiple comparison test.

To better characterize the inhibitory effect of *Ps*d1 on the colonization of pulmonary metastasis in this model, animals from each group were euthanized, and their lungs were excised and stained with HE (Figure 7). Histological analysis revealed the presence of inflammatory cells around the blood vessels and bronchi of the lung and evidence of fibrotic lesions in animals injected with B16F10 cells only (Figure 7A, red head arrows and red arrows, respectively). This profile was almost absent in animals that received just PBS (–B16F10, –Psd1), indicating that accumulation of these cells, as well as fibrosis, was related to the presence of the tumor cells only. In accordance with the observed reduction in lung tumor nodule metastasis (Figure 5A), animals co-inoculated with B16F10 cells and 1 and 3 mg/kg *Ps*d1 (+B16F10, +1 mg/kg *Ps*d1, and +B16F10, +3 mg/kg *Ps*d1) showed a great reduction in the accumulation of inflammatory cells (Figure 6A). The quantification of inflammatory foci (± SEM) per mm^2^ was obtained in six fields, corresponding to 90% of the tissue as shown in Figure 7B. When compared with animals that received just B16F10, animals co-inoculated with B16F10 cells plus 1 and 3 mg/kg *Ps*d1 (+B16F10, +1 mg/kg *Ps*d1 and +B16F10, +3 mg/kg *Ps*d1) showed a significant reduction in the accumulation of inflammatory cells (***, *p* < 0.001). We were also able to show that the injection of *Ps*d1 alone (+ 1 mg/kg *Ps*d1) provoked a very low inflammatory foci compared with animals that received B16F10 cells (*** *p* < 0.001), as observed in PBS control animals (−B16F10, −*Ps*d1). This was an important result considering the potential mammalian use for *Ps*d1. Together, these results indicated that *Ps*d1 directly suppresses the lung metastasis of circulating B16F10 melanoma cells in vivo.

## 3. Discussion

Melanoma is a very aggressive metastatic cancer that results in a quick death. Treatments based on multiple combined therapies are not effective, and the ten-year survival rate of melanoma with distant metastasis that is lower than 10% [53].

In this work, we suggest that the pea defensin *Ps*d1 could be a promising drug candidate for lung melanoma metastasis treatment using the syngeneic murine metastasis model of B16F10 melanoma cells.

In vitro data showed that the plant defensin *Ps*d1 was able to inhibit both A-431 and B16F10 cancer cell growth in a concentration-dependent manner without promoting damage to healthy cell lineages. No cell death was observed in Beas-2B human bronchial epithelial cells, where B16F10 melanoma metastasis can occur, or in the other immortalized healthy cells tested, such as HEK, R8, HSP, and CHO, suggesting that *Ps*d1 could be safe for humans.

Few works have described the inhibitory effect of plant defensins on tumor cells in vitro.The group of T.B. Ng reported that some plant defensins from the Fabaceae family showed anticancer activity and repressed the growth of leukemia and breast cancer cells [54,55,56]. However, lunatusin isolated from lima beans (*Phaseolus lunatus* L.) showed cytotoxic effects towards normal cell types and tissues [55]. They also identified that plant defensins from *Phaseolus vulgaris* and *P.coccineus* had great potential to inhibit the multiplication of colon and breast cancer cell lines without exhibiting any cytotoxic effects on normal cell types [57,58,59]. Without any effect on immortalized bovine endothelial cells, the complete inhibition of HeLa cell viability was achieved by γ-thionin defensin from *Capsicum chinense* [60]. As far as we know, none of these defensins have been tested in animal models, and in general, the mechanism of their anticancer activity has been poorly elucidated.

To gain insights into the antitumor mechanism of *Ps*d1, cell integrity studies showed that *Ps*d1 defeats the barrier function of the plasma membrane of B16F10 cells, allowing SYTOXGreen input to bind to nuclear DNA. In addition, permeation assays revealed the presence of mitochondrial oxidative stress. In this case, MitoTracker fluorescence decreased throughout *Ps*d1 treatment, indicating a reduction in the mitochondrial potential membrane [61]. After 30 min, FITC-*Ps*d1 was detected in the nuclei of DAPI-stained B16F10 cancer cells, as seen by orthogonal confocal microscopy analysis.

The signaling order of the events provoked by *Ps*d1 in B16F10 cells is not yet known, but some hypotheses may be formulated by these results. The intrinsic pathway of apoptosis is a well-established mechanism and can be activated by DNA damage via p53 protein activation or by metabolic stress. This latter can cause an increase in reactive oxygen species and a consequent loss in mitochondrial electrical potential with cytochrome C release and the activation of caspases [62]. Strong evidence has postulated that therapeutic agents that can induce ROS-mediated apoptosis in cancer cells are considered potential anticancer agents. Further studies must be carried out to determine the production level of ROS in this situation.

Some lipids and their metabolites are also involved in apoptosis, inflammation, angiogenesis, and cell proliferation signaling [63]. It is known that several cancer cell types suffer lipid metabolism reprogramming when compared to non-cancerous cells [27,36,64]. As an example, phosphatidylserine (PS), before being present in the membrane inner leaflet, is externalized [65], and cholesterol synthesis increases [27,66,67]. These changes result in a negative net charge on the cancer cell membrane, contrary to a neutral surface charge found on non-cancerous cell membranes [68,69,70].

Altered GSL metabolism leads to an upregulation of enzymes involved in this pathway, such as glucosylceramide synthase, which catalyses the transfer of a glucose residue from uridine 5′-diphospho-glucose (UDP-glucose) to the ceramide moiety [31,71,72]. The increase in GlcCer levels on cancer cell membranes has been associated with multidrug-resistant cancer cells [31,32,73] and has been proposed as a potential biomarker to evaluate the malignancy level of breast tumors [64].

An important dependence on glucosylceramide for *Ps*d1 entrance into B16F10 cell membranes was reported in this work, as detected in cells treated with PPMP, an inhibitor of glucosylceramide synthase.

Previous work by our group showed that GlcCer and ergosterol are very important for the initial interaction of *Ps*d1 with *C. albicans* [8] and *A. nidulans* [11] membranes. Mutants lacking glucosylceramide synthase are partially resistant to *Ps*d1 antifungal activity and permeabilization [8,11,15]. *Ps*d1 chemical shifts and dynamic property alterations were detected by NMR structural analysis in the presence of vesicles composed of phosphatidylcholine and GlcCer (POPC:GlcCer 90:10) [15]. The most sensitive regions in the peptide are the Gly12 and His36–Trp38 residues belonging to loop 1 and turn 3. As seen in the present work, the conserved Gly12 residue in the plant defensin family was also important for the in vitro death of B16F10 cells.

In 2007, Lobo and co-workers showed by yeast double hybrid and pull down assays that *Ps*d1 interacted with cyclin F from *N. crassa* [18]. At this time, we proposed that this phenomenon could be in part responsible for the occurrence of the *N. crassa* endoreduplication observed.

In the present work, we were able to confirm the interaction of these two proteins by surface plasmon resonance and in silico molecular docking simulations. *Ps*d1 fit well in a cavity formed between the F-box and cyclin domains of human cyclin F. Several hydrogen bonds and hydrophobic contacts were important for the maintenance of this complex. In fact, it has been proposed that disulfide bonds and polar contacts are the main forces responsible for defensin CSαβ folding stability, enabling the surface exposure of hydrophobic residues [74]. Recently, we showed that this was also valid for *Ps*d2, a pea defensin that shares 42% identity and high 3D structural homology with *Ps*d1 [6]. All hydrophobic residues are exposed on the surface, except for Leu6. They are clustered on the surface formed by two loops, between β1 and the α-helix and between β-sheets 2 and 3. We believe that these contacts between *Ps*d1 and cyclin F can disturb the interaction of cyclin F with their endogenous substrates, some of which are related to cancer diseases [75].

Cyclin F expression is enhanced in the final G2 phase, where it controls genome integrity, cell proliferation, fitness, and transcription. It is a cyclin-dependent kinase CDK-independent cyclin that contains an F-box domain and is a member of the modules of SKP1-Cullin 1-F-box protein, SCF, and E3 ligase complexes [76,77]. Cyclin F controls the ubiquitination and subsequent proteasomal degradation [20] of several target substrates, such as CP110 [78], Nusap1 [79], RRM2 [19], Cdc6 [80], DNA exonuclease Exo1 [81], SLBP [82], and, as shown very recently, the three activators E2F1, E2F2, and E2F3A of the E2F family of transcription factors [22]. In the latter case, E2F1, E2F2, and E2F3A interact with the cyclin box of cyclin F via their conserved N-terminal cyclin binding motifs.

Together, our findings suggest that the interaction of *Ps*d1 with the Fbox and the CY substrate recruitment domains of cyclin F could potentially inhibit the formation of the SCF ubiquitin–ligase complex, restricting the degradation of several substrates as E2F transcription factors. Failure to degrade E2F1, E2F2, or E2F3A in the late S and G2 phases maintains E2F activity. This, in turn, results in an imbalance of the transcriptional landscape in the G2 and M phases and in unscheduled DNA synthesis in the next cell cycle, which is accompanied by DNA replication stress and DNA damage. This is in line with the evidence that DNA replication stress can be caused by overexpression of oncoproteins [83,84] and that the control of the RB-E2F pathway is important for safeguarding genomic stability.

The capacity of *Ps*d1 to inhibit B16F10 cell growth was also tested in an in vivo model of metastatic melanoma. We reported for the first time anti-metastatic activity of a plant defensin. *Ps*d1 completely inhibited the formation of B16F10 lung metastasis nodules in mice at concentrations above 0.5 mg/kg when administered intravenously. Moreover, this effect was accompanied by the absence of weight loss in animals that received B16F10 cells plus *Ps*d1, similar to the weight pattern observed for animals that received PBS. Important weight loss was observed in animals that received only B16F10 cells, which is consistent with metastatic lung nodule implementation.

It is well known that TNF-α can be produced by alveolar macrophages. It can promote the formation and proliferation of tumors through multiple signaling pathways, and it can also promote the formation of tumor neovascularization by promoting the stable expression of interleukin 8 IL-8. IL-8 can promote the proliferation of vascular endothelial cells and can activate G proteins so that the vascular endothelial cells undergo retraction and increase the cell gap, providing conditions for tumor cell metastasis and infection [85]. The activity of *Ps*d1 was confirmed by a histological investigation that demonstrated massive infiltration into the surrounding lung parenchyma in mice that received only B16F10 cells. Infiltrating cells were not observed in animals that just received PBS and were statistically diminished when *Ps*d1 was present, confirming its therapeutic effect.

Antimicrobial peptides are only beginning to encroach into the oncological sphere, and therefore efficacy data are relatively limited [86]. In addition to its dual effect in cancer cells, the human cathelicidin LL37 is in the ongoing phase I oncology trial NCT02225366. It is being administered intratumorally in patients with documented metastatic melanoma with at least three cutaneous lesions measuring with stage IIIB, IIIC, or IV or nodal lesions. Its optimal biological therapeutic dose against metastatic melanoma is being determined in this setting. To date, no results have been published.

The safety data already obtained for AMPs in infectious disease trials substantiate the notion that AMPs, such as the pea defensin *Ps*d1, could also be well tolerated in cancer patients.

## 4. Materials and Methods

### 4.1. Expression and Purification of Psd1 and the Site-Directed Mutant Psd1 Gly12Glu

*Ps*d1 (PDB accession number 1JKZ) and the Gly12 mutant were expressed and purified as previously described [11], except that a HiPrep 26/60 Sephacryl S-100 HR column (GE Healthcare, Amersham, UK) was used in the first purification step. The purified fractions were collected, dried, and solubilized in milliQ water, and the peptide concentration was estimated using Lowry’s method [87]. The corrected amino acid primary sequences of *Ps*d1 and *Ps*d1 Gly12Glu were confirmed by LC/MS–MS analysis after peptide trypsin digestion with coverage of approximately 90%. In all cases, peptide fragments not directly related to the protein sequences of interest were not detected. LC/MS/MS analysis was performed at the CEMBIO facility (Centro de Espectrometria de Massas de Biomoléculas) at the Biophysics Federal Institute Carlos Chagas Filho at the Federal University of Rio de Janeiro.

### 4.2. Cell Lines and Culture Conditions

Murine skin melanoma (B16F10), human epidermoid carcinoma (A-431), HEK 293 (human embryonic kidney), R8 (rat lymphocyte), HSP (human hepatocyte), and CHO (Chinese hamster ovary) cell lines were maintained with Dulbecco’s Modified Eagle’s Medium - high glucose The bronchial human respiratory epithelial (Beas-2B) cell line was cultured in Roswell Park Memorial Institute Medium RPMI 1640. All media were supplemented with 10% fetal calf serum (FCS) and a solution of 100 U/mL penicillin, 100 µg/mL streptomycin, and 0.25 µg/mL Fungizone^®^ antimycotic. All cell culture reagents (unless indicated) were purchased from Thermo Fisher Scientific Inc, Maryland, USA). The cells were routinely maintained in a humidified 5% CO_2_ air incubator at 37 °C and sub-cultured every 3–4 days.

### 4.3. Cell Viability Assays

A-431, B16F10 and Beas-2B cells were seeded at a density of 1.5 × 10^4^ cells/well in a 96-well plate. After overnight incubation at 37 °C, the cells were treated in octuplicate with *Ps*d1 or *Ps*d1 Gly12Glu (0, 3.12, 6.25, 12.5, 25, or 50 µM) for an additional 24, 48, or 72 h, as indicated in each figure. HSP-2 was incubated with 6 and 30 µM *Ps*d1 for 3 h. HEK-293 for was incubated for 3 h at the same time. The cells were then washed twice with PBS (pH 7.4) and incubated with 5 mg/mL MTT (3-(4,5-dimethylthiazol-2-yl)-2,5-diphenyltetrazolium bromide) solution for 3 h. The cells were subsequently washed, and the formazan crystals formed were solubilized in DMSO. The optical density of each well was measured at a wavelength of 490 nm with an ELISA plate reader (UVM340-ASYS, Biochrom, Cambridge, UK). The effect of *Ps*d1 on cell growth was assessed as the percent of cell viability calculated by the absorbance of the cells in culture media (100% viability) and those treated with 0.1% Triton X-100 (0% viability) × 100. R8 (rat lymphocyte) and CHO (hamster ovary) cell lines were incubated with 6 and 30 µM *Ps*d1 for 3 h. Cell viability was detected by lactate dehydrogenase (LDH) release into cell culture medium through a colorimetric assay. Values are the mean ± SEM of two experiments performed in triplicate. The percent of cell viability was calculated by the absorbance of the cells in culture media (100% viability). Two independent experiments were performed in triplicate, and the values are expressed as ± standard error of the mean (SEM) after statistical analysis using one-way ANOVA with Dunnett’s multiple comparison test. *** *p* < 0.0001.

### 4.4. Confocal Microscopy Scanning

Images were obtained in the X, Y, and Z planes of the image, giving the image a 3-dimensional depth (Z-stack) at 100× magnification with a Zeiss Cell Observer Yokogawa Spinning Disk confocal microscope (Cell Observed SD, Carl-Zeiss, Oberkochen, Germany) located in the Microscopia Óptica de Luz Gustavo de Oliveira Castro (Plamol) platform in Universidade Federal do Rio de Janeiro. To measure the mean fluorescence intensity (MFI), Zen Lite Blue (Carl-Zeiss, Oberkochen, Germany) software was used. On average, thirty cells per field were analyzed in fourteen fields by experiments. Then, the region of interest (ROI), specifically the nucleus and mitochondria, was tagged on the cells photographed for fluorescence analysis, which is given by the software from the intensity of the pixels. Subsequently, the statistical test was performed using GraphPad Prism version 7.4, and the values presented are averages with the corresponding standard deviations (SDs).

### 4.5. SYTOXGreen (SG) Uptake Assay

B16F10 cells were trypsinized and counted, and 1.5 × 10^4^ cells/well in DMEM containing 0.1% (*w*/*v*) bovine serum albumin BSA were plated in a cell-view glass bottom culture dish with four compartments (Thermo Fisher Scientific Inc, Maryland, USA) and then incubated at 37 °C overnight to adhere. Post-treatment, the cells were treated with 3 or 50 µM *Ps*d1 for 30 or 60 min and maintained at the same temperature. Cells treated with only water or 0.0001% H_2_O_2_ for 30 min were used as the negative and positive controls, respectively. Washes with saline were carried out between the additions of each probe followed by incubation at room temperature. The cells were incubated with 1 µL of SG, 1.5 µL of MitoTracker Red CMXRos, and 2 µL of DAPI for 10, 20, and 10 min, respectively. The fluorescent images were acquired by confocal microscopy.

### 4.6. Psd1 Localization Fluorescence Assays

B16F10 cells (1.5 × 10^4^ cells/well) in DMEM containing 0.1% (*w*/*v*) BSA were plated in the glass-bottom of 4-well plates and then incubated at 37 °C overnight to adhere. For analysis of colocalization, MitoTracker Red CMXRos and DAPI were used to mark the mitochondria and nuclei, respectively, of live B16F10 cells in saline solution at 37 °C. The internalization of FITC-conjugated *Ps*d1 was monitored in real time for 2 h by confocal microscopy. When tested by MTT assay (see Section 4.5), 25 µM of FITC-*Ps*d1 decreased cell viability of B16F10 about 30%, a compatible value with the unlabeled peptide. Fluorescence quantification of the ROI is described in the Section 4.4 on confocal microscopy.

### 4.7. Glucosylceramide Depletion Studies on Psd1 B16F10 Cell Entrance

B16F10 cells (1.5 × 10^4^) in cover glass were previously incubated with 20 µM DL-threo-1-phenyl-2-palmitoylamino-3-morpholino-1-propanol (PPMP, Sigma-Aldrich Brasil, São Paulo, Brazil) inhibitor for 60 min followed by treatment with 9 µM *Ps*d1 for 30 min or 2 h. The cells were fixed on glass slides with 4% paraformaldehyde plus 4% sucrose for 10 min and then stained with a Vybrant Alexa Fluor 594 Lipid Raft Labeling Kit (Thermo Fisher Scientific Inc, Maryland, USA). Briefly, 1.25 µg/mL cholera toxin subunit B (CT-B) and 160× diluted anti-CT-B were incubated with cells for 10 min each at 4 °C. Three PBS wash steps were performed between each stage. PPMP-treated cell slides were mounted with ProLong Gold antifade reagent with DAPI (Life Technologies, Carlsbad, CA, USA).

### 4.8. Flow Cytometry-Based Apoptosis Detection

B16F10 cells (4 × 10^5^ cells) were treated with 50 μM *Ps*d1 for 24 h. After treatment, the cells were trypsinized, centrifuged, and washed twice with phosphate-buffered saline (PBS). The cells were then stained with Nicoletti buffer (0.1% sodium citrate, 0.1% NP-40, 200 μg/mL RNase, and 50 μg/mL propidium iodide). Doublets and debris were identified and excluded. Analysis of the DNA content was performed by collecting 20,000 events using a BD FACSCalibur flow cytometer (BD Biosciences, San Jose, CA, USA). Cells with fragmented DNA (sub-G0/G1 peak) were considered apoptotic cells.

### 4.9. Surface Plasmon Resonance Studies

Surface plasmon resonance (SPR) assays were run on a Biacore X (GE Healthcare Life Sciences, Amersham, UK) apparatus in real time using a CM5 sensor chip at 25 °C. Briefly, CM5 chip activation was performed by injection of 100 µL of a 1:1 mixture of amine coupling kit (750 mg 1-ethyl-3-(3-dimethylaminopropyl) carbodiimide hydrochloride (EDC), 115 mg *N*-hydroxysuccinimide (NHS), 2 × 10.5 mL 1.0 M ethanolamine-HCl pH 8.5) with a continuous flow of 2 µL/min. Then, 100 µL of 10 µg/µL recombinant cyclin F-GST diluted in 10 mM sodium acetate pH 4.0 buffer was immobilized by amine coupling onto the carboxylate dextran layer of CM5 with the same flow. This was injected with 80 µL of 1 M ethanolamine pH 8.0 to block the remaining binding free sites of the protein. To evaluate the interaction of *Ps*d1 with the cyclin F protein, increasing concentrations of the peptide (10–240 µM) in 100 µL of running buffer (10 mM HEPES, 150 mM NaCl, pH 7.4) were injected using a flow rate of 15 µL/min for 4 min. After the injection ended, the dissociation phase was measured in not less than 300 s. The *Ps*d1-GST-cyclin F interaction curves were subtracted from the respective curves obtained for *Ps*d1-GST to discount possible artifact interactions with GST alone. The sensorgrams obtained for each peptide–cyclin F interaction were processed by curve fitting with numerical integration analysis using BIA evaluation software 3.0.1. All analyses were run in at least duplicate.

### 4.10. Psd1–Cyclin F Molecular Docking

The ClusPro server (https://cluspro.org) was used for a blind docking simulation between *Ps*d1 and cyclin F [88]. The *Ps*d1 structure was obtained from the RCSB PDB database (code 1JKZ), while the structure of cyclin F from *Neurospora crassa* was built using the homology modeling program MODELLER. The human F-box/WD-repeat protein (code 1P22) was used as a template model, with its sequence showing 35% identity and 52% similarity with the sequence of *N. crassa* cyclin F. Only the interval between the residues Leu136 and Lys683, consisting of 548 residues out of 1010, was successfully built and used in the following computational studies. The F-box (Leu136 to Tyr177) and WD (Glu372 to Gln622) cyclin F domains were included. Every other residue outside of this interval was excluded in the final structure due to a lack of homologous templates. After these preliminary steps, both molecules (in PDB format) were sent as inputs to ClusPro selected for the advanced option “Others mode”, an algorithm that usually yields better results for protein complexes not classified as enzyme–substrate/inhibitor or antibody–antigen complexes. After docking, the two most populated structural clusters were assessed, with the selection of one central structure of each for further analysis. Post-docking analyses used the PyMOL program for generation of the molecular images, determination of the interaction interface, and detection and quantification of the intermolecular hydrogen bonds. The PSAIA (Protein Structure and Interaction Analyzer) program was used for the identification and counting of ionic, hydrophobic and van der Waals contacts. Both the interface and the intermolecular forces were defined by geometric criteria.

### 4.11. Experimental Animals

Eight-week-old C57BL/6 female mice (CEMIB, Campinas–Brasil) weighing approximately 20 g were individually marked and separated according to their experimental groups. These animals were kept in cages 20 cm × 35 cm × 15 cm (width × length × height) with free access to water and food under day and night cycles that lasted twelve hours with a controlled temperature (20–25 °C) in our own facilities. This study was approved by the Ethics Committee on the Use of Animals of Health Science Centre of the Federal University of Rio de Janeiro, Brazil (CEUA/CCS/UFRJ, CONCEA registered number 01200.001568/2013.87, approved protocol IBCCF 163 at 28 August 2012). All animals received humane care in compliance with the “Principles of Laboratory Animal Care” formulated by the National Society for Medical Research and the “Guide for the Care and Use of Laboratory Animals” prepared by the National Academy of Sciences, USA, and the National Council for Controlling Animal Experimentation, Ministry of Science, Technology and Innovation (CONCEA/MCTI), Brazil.

### 4.12. Experimental B16F10 Melanoma Metastasis Assays

Mice were injected via the lateral tail vein with 100 µL of PBS only (137 mM NaCl, 2.7 mM KCl, 10 mM Na_2_HPO_4_, 2 mM KH_2_PO_4_, pH 7.4) (*n* = 17) or 100 µL of B16F10 cells (2.5 × 10^5^ cells/animal suspended in DMEM) (*n* = 15). Other groups of animals were intravenously co-injected with a freshly prepared, in order to avoid B16F10 cells death, mix of B16F10 cells plus 0.1 mg/kg (*n* = 8), 0.5 mg/kg (*n* = 8), 1 mg/kg (*n* = 17), or 3 mg/kg *Ps*d1 (*n* = 7).

Another group of animals received 1 mg/kg of *Ps*d1 only (*n* = 9) to evaluate *Ps*d1 toxicity. Twenty-one days later, the animals were anaesthetized and sacrificed, and the black lung melanoma nodules were counted and measured under a dissecting microscope (Zeiss AxioPhot fluorescence microscope (Carl-Zeiss, Oberkochen, Germany)).

### 4.13. Histological Analysis

After the above procedure, two animals from each group were anaesthetized and submitted to treatment with 4% paraformaldehyde in 0.1 M phosphate buffer (pH 7.4) by transcardial perfusion. The lung was removed and immersed in the same fixative solution. The lung specimens were sliced into 5 mm pieces, dehydrated through an ascending ethanol series (70, 95, and 100% ethanol for 30 min each) and then embedded in paraffin using standard procedures. Serial 5 µm-thick sections were prepared using a sliding microtome (Leica Microsystems, Wetzlar, Germany). Hematoxylin and eosin (HE) staining wereperformed to quantify possible foci of inflammatory infiltrates and fibrosis in experimental and control animals. The stained sections were observed, and digital images were taken with a Zeiss AxioPhot fluorescence microscope (Carl-Zeiss, Oberkochen, Germany). Specifically, two slices of each sample (six fields of each slice corresponding to approximately 90% of the slice) were used for quantitative analysis to obtain the mean value.

### 4.14. Data Analysis

The results are presented as the mean values ± SD and were interpreted using one-way ANOVA with Dunnett’s or Bonferroni post-tests or by two-way ANOVA, according to each experiment and as indicated in each figure legend. Differences were statistically significant when the *p* value was less than 0.05.

## Supplementary Materials

Supplementary materials can be found at https://www.mdpi.com/1422-0067/21/8/2662/s1.
