# Supplementary material for: Pisum sativum Defensin 1 Eradicates Mouse Metastatic Lung Nodules from B16F10 Melanoma Cells
Source: Int J Mol Sci. 2020 Apr 11;21(8):2662. doi: 10.3390/ijms21082662 (PMC7219108; doi:10.3390/ijms21082662)
Supplement: Supplementary file 1 [file ijms-21-02662-s001.zip › Supplementarytable1ijms-713239 (1).pdf]

Table S1. Non-covalent interactions between *Ps*d1 and cyclin F. Legend for types of interactions: HB, Hydrogen bond; EA, Electrostactic attraction; HC, Hydrophobic contact and VDW, Van der Waals interaction.

*Ps*d1 residue	Cyclin F residue	Interaction Type
Leu6	Tyr177	HB, VDW
Arg11	Lys470	HB, VDW
Gly12	Lys470	HB, VDW
Ala28	Lys171	HB, VDW
His29	Tyr147	HB, VDW
Ile31	Tyr147	HB, VDW
Cys35	Arg546	HB, VDW
Trp38	Ile543	HB, VDW
Trp38	Glu545	HB, VDW
Lys39	Pro233	HB, VDW
Gln44	His175	HB, VDW
Asn45	Tyr147	HB, VDW
Arg11	Glu275	2 HBs, EA, VDW
Arg11	Glu429	EA
Lys39	Asp235	HB, EA, VDW
Leu6	Pro230	HC, VDW
Leu6	Pro233	HC, VDW
Val13	Ile472	HC, VDW
Phe15	Ile472	HC, VDW
Phe15	Pro563	HC, VDW
Cys3, His5, Ala7, Asp8, Arg11, Cys14, Thr16, Ala28, Leu30, Cys35, His36, Asn37, Trp38, Lys39, Thr43, Gln44, Asn45, Cys46	Leu145, Gly146, Tyr147, Leu148, Tyr177, Tyr178, Met179, Glu180, Ala234, Met273, Asp469, Lys470, Arg471, Arg487, His544, Glu545, Arg546, Trp562, Pro563, Gly585, Arg586	VDW
